# Bioactive Azafluorenone Alkaloids from *Polyalthia debilis* (Pierre) Finet & Gagnep

**DOI:** 10.3390/molecules14114414

**Published:** 2009-11-03

**Authors:** Supaluk Prachayasittikul, Patumporn Manam, Maneekarn Chinworrungsee, Chartchalerm Isarankura-Na-Ayudhya, Somsak Ruchirawat, Virapong Prachayasittikul

**Affiliations:** 1 Department of Chemistry, Faculty of Science, Srinakharinwirot University, Bangkok 10110, Thailand; 2 Department of Clinical Microbiology, Faculty of Medical Technology, Mahidol University, Bangkok 10700 , Thailand; 3 Chulabhorn Research Institute and Chulabhorn Graduate Institute, Bangkok 10210, Thailand

**Keywords:** *Polyalthia debilis*, azafluorenone, onychine, antimicrobial, antimalarial and cytotoxic activities

## Abstract

This study investigated bioactive extracts of *Polyalthia debilis* (Annonaceae) with antimicrobial, antimalarial and cytotoxic activities. Extensive chromatographic isolations provided azafluorenone alkaloids; onychine (**1**) and 7-methoxyonychine (**2**) together with a mixture of *β*–sitosterol and stigmasterol. The two alkaloids were isolated from the *P. debilis* for the first time. Isolated fractions containing a mixture of triterpenoids (C7, C8 and C9) exhibited the most potent antimicrobial activity against many bacterial strains with minimum inhibitory concentration of 64 μg/mL. Fractions with antimalarial and cytotoxic activities were also observed. The findings suggest the potential use of *P. debilis* in medicinal applications.

## Introduction

*Polyalthia debilis* (Pierre) FINET & GAGNEP. (Annonaceae), is a Thai medicinal plant whose root water decoction has been traditionally used for treatment of abdominal pain and tuberculosis, as a febrifuge and a galactogogue [1]. Previously, isolation of some new antimalarial dimeric aporphines from the root of *P. debilis* was reported [2]. To identify additional and new bioactive compounds, the present study investigated the isolation, antimicrobial, antimalarial and cytotoxic activities of chloroform and ethyl acetate extracts of *P. debilis*.

## Results and Discussion

### Isolation and structure elucidation

**Figure 1:**
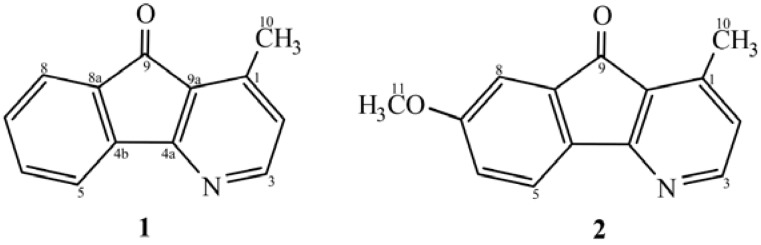
Chemical structures and numbering of alkaloids **1** and **2**.

Chloroform (PC) and ethyl acetate (PE) extracts of the root of *P. debilis* were tested for antimicrobial and anticancer activities. Bioassay guided isolation was performed by repeated silica gel column chromatography using gradient elution with increasing polarity. Starting from 6 kg of the dried roots, the PC extract provided two azafluorenone alkaloids; 1-methyl-4-azafluoren-9-one (1.5 mg onychine, **1**) and 7-methoxy-1-methyl-4-azafluoren-9-one (**2**, 1.7 mg) from fractions C6.6.2 and C6.6.3, respectively. In addition, a mixture of *β*-sitosterol and stigmasterol was isolated from fractions C3.3, C3.5 and C5. Extensive isolation of the PE extract gave a mixture of *β*-sitosterol and stigmasterol from fraction E3.3. The alkaloids **1** and **2** were not previously reported to be isolated from *P. debilis*. Onychine, (**1**, 90 mg) was first isolated from the trunkwood (12 kg) of a Brazilian Annonaceae, *Onychopetalum amazonicum* [3]. Later **1** was isolated from other plant species; 650 mg from the root bark (500 g) of an African Annonaceae, *Cleistopholis patens* [4], from the bark (2.75 kg) of *P. longifolia* [5], 15 mg from the trunk bark (1.5 kg) of *Unonopsis spectabilis* [6] and 4 mg from the trunk wood (7 kg) of *Guatteria dielsiana* [7]. The onychine derivative **2** was found in the plant species *Porcelia macrocarpa* [8,9]. It is obvious that the amounts of alkaloids **1** and **2** found in *P. debilis* werevery small compared with the other plant species. Structures of the isolates **1** and **2** (Figure 1) were confirmed by comparison of their spectral data; UV, IR, ^1^H- and ^13^C-NMR with the literature data. 2D-NMR (COSY, HMQC, HMBC, DEPT90 and DEPT135) were also performed in this study. In 1976 the structure of natural onychine was assigned based on its UV, IR, ^1^H-NMR and MS spectra as 1-aza-4-methyl-fluoren-9-one [3]. Later the synthesis of onychine was reported [10,11] and its structure was revised to 4-aza-1-methyl-fluoren-9-one [10]. To elucidate core structure of the revised azafluorenone, therefore, a series of related derivatives bearing methoxy group at 5-, 6-, 7- and 8-positions including 6,7-dimethoxyonychine was synthesized [12,13]. Their structures were fully established by the aid of heteronuclear 2D-NMR; a long-range correlation of ^1^H-^13^C(COLOG) and COSY spectra [13]. It was observed that H-8 (δ7.58 ppm) was correlated with quaternary carbons; C-9(CO, δ 192.8 ppm) and C4b (δ 142.7 ppm). Similarly, the correlation between H-6 and C-4b was also noted [13]. In our HMBC spectra of compounds **1** and **2** reveal similar ^1^H-^13^C correlations such as CH_3_ with C-1, C-2, C-9(C-9a); H-2 with C-3, C-9, C-9a; H-3 with C-4a, C-1, C-2. Particularly for 7-methoxyonychine (**2**), its OCH_3_ (δ3.91 ppm) was correlated with C-7(δ162.6 ppm).

### Biological activities

The *P. debilis* extracts (PC and PE) and their fractions (C2-C9, E3.6, E4 and E5) were tested for bioactivity.

### Antimicrobial activity

This activity was tested against 27 strains of microorganisms using the agar dilution method [14]. Results (Table 1) showed that except for fractions C6 and E5 that exhibited partial growth inhibition, the tested extracts and fractions displayed complete antigrowth activity against many microorganisms with MIC values ranging from 64-256 μg/mL. It is interesting to note that fractions C7, C8 and C9 from the chloroform extract (PC) displayed the highest antimicrobial activity against *Corynebacterium diphtheriae* NCTC 10356, *Bacillus subtilis* ATCC 6633 and *Bacillus cereus* with MICs of 64 μg/mL, while the extracts (PC and PE) and fraction E4 from PE showed antigrowth activity against such organisms with MICs of 256 μg/mL. The aforementioned organisms were also inhibited by fraction C5, with MICs of 128-256 μg/mL. In addition, the fractions containing a mixture of triterpenoids (C7, C8 and C9) also inhibited the growth of *Branhamella catarrhalis* with a MIC of 64 μg/mL. At the MIC of 256 μg/mL fraction C2 selectively showed antigrowth activity agains *B. subtilis* ATCC 6633, while E3.6 selectively inhibited the growth of *C. diphtheriae* NCTC 10356. Fraction C3 (against *C. diphtheriae* NCTC 10356), C7 (against *Plesiomonas shigelloides*) and C8 (against *Micrococcus lutens* ATCC 10240, *P. shigelloides* and *Streptococcus pyogenes*) exerted potent antimicrobials with MIC of 64 μg/mL. Complete inhibition of *Staphylococcus epidermidis* ATCC 12228 and *Enterococcus faecalis* ATCC 29212 were observed from fractions C8 and C9 with MIC of 256 μg/mL. *Staphylococcus aureus* ATCC 25923 was inhibited by the fraction C8 with MIC of 256 μg/mL. Moreover, C8 was the only component that showed antifungal activity against *Candida albicans* (75% inhibition) at 256 μg/mL. Unfortunately, the isolates **1** and **2** were not evaluated for antimicrobials, due to the limited quantity of the compounds available. Onychine (**1**) was shown to be active against *C. albicans* B311 with MIC of 3.12 μg/mL [15]. In addition, compound **1** also exhibited antimicrobial activity against many organisms e.g. *S. aureus* NCTC 8530, *B. subtilis* IFO 3007, *Escherichia coli* IFO 3545 and *Saccharomyces cerevisiae* IFO 0203 with MIC range 50 to >100 μg/mL [16]. To date, the antimicrobial activity of *P. debilis* was not reported in the literature. Previously, a mixture of 6- and 7-methoxyonychines was reported to show a weak DNA-damaging potential [9].

**Table 1:** Antimicrobial activity* of *P. debilis*.

Compound ^a,b,c^	Microorganism	MIC ^e^ (µg/mL)
PC	*C. diphtheriae* NCTC 10356, *B. subtilis* ATCC 6633,	256
	*B. cereus*, *B. catarrhalis*, *P. shigelloides*	
PE	*C. diphtheriae* NCTC 10356, *B. subtilis* ATCC 6633,	256
	*B. cereus*	
C2	*B. subtilis* ATCC 6633	256
C3	*C. diphtheriae* NCTC 10356,	64
	*B. subtilis* ATCC 6633, *B. catarrhalis*	128
	*M. lutens* ATCC 10240, *B. cereus*, *P. shigelloides*, *M. flavas*	256
C4	*C. diphtheriae* NCTC 10356, *B. subtilis* ATCC 6633	256
C5	*C. diphtheriae* NCTC 10356, *B. subtilis* ATCC 6633	128
	*B. cereus*	256
C7^d^	*C. diphtheriae* NCTC 10356, *B. subtilis* ATCC 6633,	64
	*B. cereus*, *B. catarrhalis*, *P. shigelloides*	
	*S. pyogenes*	256
C8^d^	*C. diphtheriae* NCTC 10356, *B. subtilis* ATCC 6633,	64
	*M. lutens* ATCC 10240, *B. cereus*, *B. catarrhalis*, *P. shigelloides*, *S. pyogenes*	
	*S. aureus* ATCC 25923, *S. epidermidis* ATCC 12228,	256
	*E. faecalis* ATCC 29212, *M. flavas*, *E. tarda*, *N. mucosa, L. monocytogenes*	
C9^d^	*C. diphtheriae* NCTC 10356, *B. subtilis* ATCC 6633, *B. cereus*, *B. catarrhalis*	64
	*P. shigelloides*	128
	*M. lutens* ATCC 10240, *S. epidermidis* ATCC 12228, *E. faecalis* ATCC 29212, *E. tarda*	256
E3.6	*C. diphtheriae* NCTC 10356	256
E4	*C. diphtheriae* NCTC 10356, *B. subtilis* ATCC 6633, *B. cereus*, *B. catarrhalis*, *P. shigelloides*	256

Partial inhibition at 256 μg/mL: ^a ^C6 against *C. diphtheriae* NCTC 10356 (50%), *S. pyogenes* (75%); ^b ^C8 against *C. albicans* (75%); ^c ^E5 against *C. diphtheriae* NCTC 10356 and *B. subtilis* ATCC 6633 (50%). *Ampicillin at 10 μg/mL showed complete inhibition against *S. aureus* ATCC 25923, *S. epidermidis* ATCC 12228, *B. subtilis* ATCC 6633, *N. mucosa*, *B. catarrhalis*, *E. tarda* and *S. pyogenes*. ^d ^Fractions contained a mixture of triterpenoids. ^e^ minimum inhibitory concentration.

### Antimalarial activity

Antimalarial activity of the extracts and fractions was tested [17] against chloroquine resistant *Plasmodium falciparum* (T9.94). It was revealed (Table 2) that most of the tested extracts (PC and PE) and fractions (C2-C9, E4 and E5) displayed fair activity with IC_50_ values of 100-1000 μg/mL. Only the fraction E3.6 showed good activity, with an IC_50_ of 10-100 μg/mL. Previously, the dichloromethane extract and isolates; dimeric aporphine alkaloids of *P. debilis* were reported to be antimalarials [2]. The alkaloids **1** and **2**, including chloroform and ethyl acetate extracts, as well as the isolated fractions of *P. debilis* were not tested for antimalarial action.

**Table 2:** Antimalarial activity of *P. debilis.*

Compound^a^	Activity	IC_50_ (µg/mL)
PC, PE	fair	100-1,000
C2-C9, E4, E5	fair	100-1,000
E3.6	good	10-100

^a^ Chloroquine hydrochloride was a standard drug.

### Cytotoxic activity

Cytotoxic assay was evaluated [18] against three cell lines. The results (Table 3) showed that the extract (PC) and many fractions (C3-C9, E3.6 and E5) exhibited cytotoxic activity against all the tested cell lines. However, ethyl acetate extract and fractions (C2 and E4) were shown to be inactive cytotoxic agents (IC_50_ > 50 μg/mL). Among the cytotoxic agents, fractions C3 and C9 exhibited the highest activity against A549 cells with comparable IC_50_ of 6.0 ± 2.8 and 6.75 ± 0.4 μg/mL, respectively. Fraction C5 displayed the most potent activity against HepG2 cells with IC_50_ of 7.0 ± 2.5 μg/mL. In addition, the C5 also showed the highest cytotoxic activity against HCC-S102 cells (IC_50_ 8.5 ± 0.7 μg/mL). So far cytotoxic activity of the *P.debilis* was not reported elsewhere.

## Conclusions

Bioactive extracts (chloroform and ethyl acetate) of *P. debilis* were investigated and found to give many fractions with antimicrobial, antimalarial and cytotoxic activities. Particularly, fractions C7, C8 and C9 displayed the most potent antimicrobial activity against many bacterial strains with MICs of 64 μg/mL. Extensive chromatographic separations afforded two azafluorenone alkaloids; onychine (**1**) and 7-methoxyonychine (**2**) together with a mixture of *β*–sitosterol and stigmasterol. In this study, the two alkaloids **1** and **2** were isolated from *P. debilis* for the first time. Compound **1** when isolated from other Annonaceae plants exhibited antibacterial and antifungal actions. The findings suggest the potential use of the *P. debilis* in medicinal applications and as a guide to synthetic chemistry in order to find new bioactive lead compounds.

**Table 3:** Cytotoxic activity of *P. debilis.*

Compound	IC_50_ (µg/mL)^a,b,c^		
	HepG2	A549	HCC-S102
PC	23.0 ± 1.4	19.0 ± 1.4	18.0 ± 1.4
PE	>50	>50	>50
C2	>50	>50	>50
C3	15.0 ± 4.2	6.0 ± 2.8	11.0 ± 1.4
C4	15.0 ± 0.0	10.7 ± 3.3	14.5 ± 2.1
C5	7.0 ± 2.5	14.5 ± 3.5	8.5 ± 0.7
C6	15.0 ± 1.4	22.5 ± 0.7	17.5 ± 2.1
C7	22.0 ± 7.1	34.5 ± 2.1	25.0 ± 2.8
C8	14.5 ± 4.9	17.5 ± 4.9	17.0 ± 1.4
C9	9.3 ± 6.6	6.75 ± 0.4	10.6 ± 1.4
E3.6	12.5 ± 0.7	18.0 ± 0.0	16.0 ± 1.4
E4	>50	>50	>50
E5	40.5 ± 7.8	>50	45.0 ± 7.1
Etoposide	0.20	0.34	0.32

^a^ Cell lines are: HepG2 Human hepatocellular liver carcinoma cell line; A549 Human lung carcinoma cell line; HCC-S102 Hepatocellular carcinoma cell line. ^b^ When IC_50_ > 50 μg/mL denotes inactive cytotoxic activity. ^c^ The assays were performed in triplicate using etoposide as the reference drug.

## Experimental

### General

Melting points were determined on an Electrothermal melting point apparatus (Electrothermal 9100) and are uncorrected. ^1^H- and ^13^C-NMR spectra were recorded on Bruker AVANCE 300 and 600 NMR spectrometers (operating at 300 and 600 MHz for ^1^H and 75 and 125 MHz for ^13^C, respectively). Infrared spectra (IR) were obtained on a Perkin Elmer System 2000 FTIR. Ultraviolet (UV) spectra were recorded on a Shimadzu UV 240 IPC. Mass spectra were recorded on a Finnigan INCOS 50 and Bruker Daltonics (micro TOF). Column chromatography was carried out using silica gel 60 (0.063–0.200 mm). Analytical thin layer chromatography (TLC) was performed on silica gel 60 PF_254_ aluminium sheets (cat. No. 7747 E., Merck). Solvents were distilled prior to use. Reagents for cell culture and assays were of analytical grade as the following: RPMI-1640 (Rosewell Park Memorial Institute medium, Gibco and Hyclone laboratories, USA), HEPES (*N*-2-hydroxyethylpiperazine-*N′*-2-ethanesulfonic acid), L-glutamine, penicillin, streptomycin, sodium pyruvate and glucose (Sigma, USA), Ham’s/F12 (Nutrient mixture F-12), DMEM (Dulbecco’s Modified Eagle’s Medium) and FBS (fetal bovine serum, Hyclone laboratories, USA), gentamicin sulfate (Government Pharmaceutical Organization, Thailand), 3(4,5-dimethylthiazol-2-yl)-2,5-diphenyltetrazolium bromide (Sigma-Aldrich, USA).

### Plant material

Roots of *P. debilis* were collected from Ubonratchatanee Province, Thailand. It has been identified (BKF 135063) by The Forest Herbarium, Royal Forestry Department, Bangkok. A voucher specimen has been deposited at Department of Chemistry, Faculty of Science, Srinakharinwirot University, Bangkok, Thailand.

### Isolation

Chloroform and ethyl acetate extracts were obtained from dried powdered roots (6 kg) of *P. debilis*. Chloroform extract (PC, 100 g) of the plant was isolated by silica gel (1,000 g) column chromatography using gradient elution with dichloromethane enriched with ethyl acetate. Fractions were collected and combined based on TLC chromatograms. Solvents were removed *in vacuo* to give nine fractions (C1-C9) of dark brown gum. Fractions C3, C5 and C6 were reseparated on a silica gel column. Fraction C3 (1.5 g from dichloromethane elution) was subjected to silica gel (25 g) column chromatography to afford seven fractions (C3.1-C3.7) of dark gum upon elution with hexane-dichloromethane, then dichloromethane-methanol. Fractions C3.3 and C3.5 were rechromatographed as described. Fraction C3.3 (259 mg) gave a mixture of *β*-sitosterol and stigmasterol (14.5 mg as white solid). Fraction C3.5 (422 mg) provided a mixture of *β*-sitosterol and stigmasterol (30.4 mg). Fraction C5 (5.1 g from dichloromethane:ethyl acetate, 9:1) on silica gel (200 g) column chromatography eluted with hexane-dichloromethane then dichloromethane-methanol gave a mixture of *β*-sitosterol and stigmasterol (6.7 mg).

Fraction C6 (17 g from dichloromethane:ethyl acetate, 8:2) was separated on a silica gel (600 g) column. Elution with hexane-dichloromethane then dichloromethane-methanol afforded seven fractions (C6.1-C6.7). Fraction C6.6 (15.1 g) was rechromatographed on silica gel (55 g). Elution with hexane-acetone gave 11 fractions of yellow oil and dark brown gum (C6.6.1-C6.6.11), the fractions eluted from hexane-acetone, 98:2 (C6.6.2 and C6.6.3) were further separated. Fraction C6.6.2 (6.6 mg of yellow oil) was chromatograhed on silica gel (1.5 g) eluting with hexane-acetone, 98:2 to give a yellow solid (1.5 mg) of compound **1** (1-methyl-4-azafluoren-9-one or onychine); mp 124-126 °C (lit mp 125-127°C [11], 125-129 °C [7], 133-135°C [10]; R_f_ = 0.95 (hexane-acetone, 9:1); UV (EtOH) λ_max_ (logε): 252 (4.16), 279 (3.87), 289 (3.88), 307 (3.42) nm; IR (UATR solid): υ_max_ 2,925, 1,702, 1,596, 1,564, 1,448, 1,371, 756 cm^-1^; ^1^H-NMR (acetone-*d_6_*): δ 2.60 (d, 3H, *J* = 0.48 Hz, H-10), 7.14 (dd, 1H, *J* = 5.26, 0.48 Hz, H-2), 7.51(td, 1H, *J* = 7.43, 1.23 Hz, H-7), 7.66 (dd, 1H, *J* = 7.43, 1.23 Hz, H-8), 7.67 (td, 1H, *J* = 7.43, 1.23 Hz, H-6), 7.84 (dd, 1H, *J* = 7.43, 1.23 Hz, H-5), 8.45 (d, 1H, *J* = 5.26 Hz, H-3); ^13^C-NMR (acetone-*d_6_*): δ 16.8 (C-10), 121.3 (C-5), 123.9 (C-8), 126.2 (C-9a), 126.6 (C-2), 131.5 (C-7), 135.5 (C-8a), 135.7 (C-6), 143.9 (C-4b), 147.9 (C-1), 153.7 (C-3), 165.6 (C-4a), 193.2 (C-9); LRMS (EI): *m/z* (%) = 195 (72) [M]^+^, 167 (9), 166 (7), 139 (6); HRMS (TOF): *m/z* [M+H]^+^ calcd. for C_13_H_10_NO:196.0761 found:196.0757. Similarly, fraction C6.6.3 (5.9 mg) on silica gel (1.5 g) was eluted by hexane-acetone, 95:5 to afford a yellow solid (1.7 mg) of compound **2** (7-methoxy-1-methyl-4-azafluoren-9-one); mp 175-178°C (lit mp 179 °C [12], R_f_ = 0.81 (hexane-acetone, 9:1); UV (EtOH) λ_max_ (logε): 264 (4.50), 293 (3.88), 312 (3.64), 326 (3.53) nm; IR (UATR solid): υ_max_ 2,923, 1,711, 1,596, 1,567, 1,290, 1,228, 797 cm^-1^; ^1^H-NMR (acetone-*d_6_*): δ 2.55 (s, 3H, H-10), 3.91 (s, 3H, H-11), 7.02(d, 1H, *J* = 5.30 Hz, H-2), 7.17 (dd, 1H, *J* = 8.89, 2.38 Hz, H-6), 7.18 (d, 1H, *J* = 2.38 Hz, H-8), 7.73 (d, 1H, *J* = 8.89 Hz, H-5), 8.35 (d, 1H, *J* = 5.30 Hz, H-3); ^13^C-NMR (acetone-*d_6_*): δ 16.1 (C-10), 55.4 (C-11), 108.8 (C-8), 120.1 (C-6), 122.0 (C-5), 124.8 (C-2), 125.4 (C-9a), 135.5 (C-4b), 136.9 (C-8a), 146.9 (C-1), 152.9 (C-3), 162.6 (C-7), 165.4 (C-4a), 192.4 (C-9); LRMS (EI): *m/z* (%) = 225 (100) [M]^+^, 210 (7), 182 (8), 154 (14); HRMS (TOF): *m/z* [M+H]^+^ calcd. for C_14_H_12_NO_2_:226.0855 found:226.0863.

The ethyl acetate extract (PE, 36.0 g) was chromatographed on a silica gel (120 g) column. Elution with gradient solvent from hexane, dichloromethane, ethyl acetate then methanol gave five fractions (E1-E5) of dark to black gum. Fraction E3 (4.0 g from hexane-dichloromethane, 4:6 elutions) was repeatedly isolated on silica gel (200 g) to give eight fractions (E3.1-E3.8). Fraction E3.3 (131.2 mg from hexane-ethyl acetate, 94:6) was further separated on silica gel (10 g), eluting with dichloromethane-ethyl acetate, 98:2 to give a mixture of *β*-sitosterol and stigmasterol (14.6 mg).

### Antimicrobial assay

Antimicrobial activity of the tested compounds was carried out using the agar dilution method [14]. Briefly, the tested compounds dissolved in DMSO were individually mixed with 1 mL Müller Hinton (MH) broth while the negative control was the MH broth with omission of the tested compounds. The solution was then transferred to the MH agar solution to yield the final concentrations of 32-256 μg/mL. Twenty seven strains of microorganisms as listed below, cultured in MH broth at 37 °C for 24 h, were diluted with 0.9 % normal saline solution to adjust the cell density of 3 × 10^9^ cell/mL. The organisms were inoculated onto each plate and further incubated at 37 °C for 18-48 h. Compounds which possessed high efficacy to inhibit bacterial cell growth were analyzed. Tested microorganisms were gram-negative bacteria: *Escherichia coli* ATCC 25922, *Klebsiella pneumoniae* ATCC 700603, *Salmonella typhimurium* ATCC 13311, *Salmonella choleraesuis* ATCC 10708, *Pseudomonas aeruginosa* ATCC 15442, *Edwardsiella tarda*, *Shigella dysenteriae*, *Citrobacter freundii*, *Morganella morganii*, *Vibrio cholera*, *Vibrio mimicus*, *Aeromonas hydrophila*, *Plesiomonas shigelloides*, *Xanthomonas maltophilia*, *Neisseria mucosa*, *Branhamella catarrhalis*, gram-positive bacteria: *Stapphylococcus aureus* ATCC 25923, *Stapphylococcus epidermidis* ATCC 12228, *Enterococcus faecalis* ATCC 29212, *Micrococcus lutens* ATCC 10240, *Corynebacterium diphtheriae* NCTC10356, *Bacillus subtilis* ATCC 6633, *Streptococcus pyogenes*, *Listeria monocytogenes*, *Bacillus cereus*, *Micrococcus flavas* and diploid fungus (yeast): *Candida albicans*.

### Antimalarial assay

Antimalarial activity of the tested compounds was evaluated against chloroquine resistant *Plasmodium falciparum* (T9.94) using the literature method [19]. Human erythrocytes (type O) infected with chloroquine resistant *P. falciparum* (T9.94) were maintained in continuous culture, according to the method described previously [17]. RPMI 1640 culture medium supplemented with 25 mM of HEPES, 40 mg/L gentamicin sulfate and 10 mL of human serum was used in continuous culture. Before performing the experiment, *P. falciparum* culture was synchronized by using sorbitol induced hemolysis according to the method of Lambros and Vanderberg [20] to obtain only ring stage-infected red blood cells and then incubated for 48 h prior to the drug testing to avoid effect of sorbitol.

The experiments were started with synchronized suspension of 0.5% to 1% infected red blood cell during ring stage. Parasites were suspended with culture medium supplemented with 15% human serum to obtain 10% cell suspension. The parasite suspension was put into 96-well microculture plate; 50 μL in each well and then add 50 μL of various tested drug concentrations. These parasite suspensions were incubated for 48 h in the atmosphere of 5% CO_2_ at 37 °C. The percents parasitemia of control and drug-treated groups were examined by microscopic technique using methanol-fixed Giemsa stained of thin smear blood preparation. The efficacy of the drugs were evaluated by determining the drug concentration that reduced parasite growth by 50% (IC_50_).

### Cytotoxic assay

Cells were grown in Ham’s/F12 medium containing 2 mM L-glutamine supplemented with 100 U/mL penicillin, streptomycin and 10% fetal bovine serum. Except HepG2 cell was grown in DMEM. Cytotoxic assay was performed using the modified method as previously described [18]. In brief, cell lines suspended in RPMI-1640 containing 10% FBS were seeded at 1 × 10^4^ cells (100 μL) per well in 96-well plate and incubated in humidified atmosphere, 95% air, 5%CO_2_ at 37 °C. After 24 h, additional medium (100 μL) containing the test compound and vehicle was added to a final concentration of 50 μg/mL, 0.2% DMSO, and further incubated for 3 days. Cells were subsequently fixed with 95% EtOH, stained with crystal violet solution, and lysed with a solution of 0.1 N HCl in MeOH, after which absorbance was measured at 550 nm. Whereas A549 and HepG2 cells were stained by MTT (3-(4,5-dimethylthiazol-2-yl)-2,5-diphenyl tetrazolium bromide). IC_50_ values were determined as the drug and sample concentrations at 50% inhibition of the cell growth.
